# NADPH Oxidases and Their Role in Atherosclerosis

**DOI:** 10.3390/biomedicines8070206

**Published:** 2020-07-10

**Authors:** Anastasia V. Poznyak, Andrey V. Grechko, Varvara A. Orekhova, Victoria Khotina, Ekaterina A. Ivanova, Alexander N. Orekhov

**Affiliations:** 1 Department of Basic Research, Institute for Atherosclerosis Research, Skolkovo Innovative Center, 121609 Moscow, Russia; tehhy_85@mail.ru (A.V.P.); kate.ivanov@gmail.com (E.A.I.); 2 Federal Research and Clinical Center of Intensive Care Medicine and Rehabilitology, 14-3 Solyanka Street, 109240 Moscow, Russia; noo@fnkcrr.ru; 3 Laboratory of Medical Genetics, National Medical Research Center of Cardiology, 15A 3-rd Cherepkovskaya Street, 121552 Moscow, Russia; varvaraao@gmail.com; 4 Laboratory of Infectious Pathology and Molecular Microecology, Institute of Human Morphology, 3 Tsyurupa Street, 117418 Moscow, Russia; nafany905@gmail.com; 5 Laboratory of Angiopathology, Institute of General Pathology and Pathophysiology, 8, Baltiyskaya St., 125315 Moscow, Russia

**Keywords:** NADPH oxidase, atherosclerosis, CAD, ROS, oxidative stress

## Abstract

The current view on atherosclerosis positions it as a multifactorial disorder that results from the interplay between lipid metabolism disturbances and inflammatory processes. Oxidative stress is proven to be one of the initiating factors in atherosclerosis development, being implicated both in the inflammatory response and in atherogenic modifications of lipoproteins that facilitate lipid accumulation in the arterial wall. The hallmark of oxidative stress is the elevated level of reactive oxygen species (ROS). Correspondingly, the activity of major ROS-generating enzymes, including nicotinamide adenine dinucleotide phosphate (NADPH) oxidases, xanthine oxidases, and cyclooxygenases, is an important element in atherosclerosis development. In particular, the role of NADPH oxidases in atherosclerosis development has become a subject of intensive research. Aberrant activity of NADPH oxidases was shown to be associated with cardiovascular disease in humans. With regard to atherosclerosis, several important pathological components of the disease development, including endothelial dysfunction, inflammation, and vascular remodeling, involve aberrations in NADPH oxidases functioning. In humans, NADPH oxidases are represented by four isoforms expressed in vascular tissues, where they serve as the main source of ROS during atherogenesis. Moreover, recent studies have demonstrated their impact on vascular remodeling processes. Interestingly, one of the NADPH oxidase isoforms, NOX4, was shown to have an atheroprotective effect. Despite the growing evidence of the crucial involvement of NADPH oxidases in atherosclerosis pathogenesis, the available data still remains controversial. In this narrative review, we summarize the current knowledge of the role of NADPH oxidases in atherosclerosis and outline the future directions of research.

## 1. Introduction

Historically, nicotinamide adenine dinucleotide phosphate (NADPH) oxidases of the NOX family were first detected in the membranes of so-called professional phagocytic cells of the immune system, such as macrophages. These cells represent the front line of innate immunity, responsible for removal of pathogens and defective host cells by internalizing them through phagocytosis and degrading with lysosomal enzymes. In such cells, NADPH oxidases are responsible for generation of superoxide needed for initial pathogen killing [1]. Later, the presence of NADPH oxidase homologs was also demonstrated in other cell types, such as fibroblasts, endothelial cells (EC), smooth muscular cells (SMC), and others. The search for homologs of the NADPH oxidase expressed by phagocytes revealed as many as six members of the newly called NOX family of NADPH oxidases: NOX1-5 and two large enzymes DUOX1 and 2. The first identified and most extensively studied member of the NOX family is NOX2. All NOX family enzymes are transmembrane proteins that contain six conserved transmembrane domains. At the cytoplasmic c-terminus, the enzymes have a NADPH-binding region, which is also highly conserved, followed by a FAD-binding site. The transmembrane domains contain four heme-binding histidines. The NOX NADPH oxidases are often heavily glycosylated. Some of the family members (DUOX1 and 2) are much larger in size than NOX2 and contain additional domains. The transmembrane location of the enzymes allows them to transport the electrons across the membrane, which is coupled with the reduction of oxygen to superoxide. That, in turn, can give rise to other reactive oxygen species (ROS) [1].

Under normal physiological conditions, the level of NADPH oxidases expression in nonphagocytic cells is rather low. However, it can be significantly increased when cells are exposed to mitogenic and/or transforming growth factors, high glucose levels, or hyperlipidemia. These signals lead to an increase in ROS production and contribute to oxidative stress development [2,3]. The extent of ROS formation by NOX enzymes is dependent on the level of their expression, and the mechanisms of expression regulation are no less important than activation of NOX through phosphorylation-dependent pathways. Epigenetic regulation of NOX expression involves DNA methylation, regulation of mRNA stability, presence of noncoding RNAs, post-translational modification of histones, and variations of activity of transcriptional factors [4].

**Figure 1:**
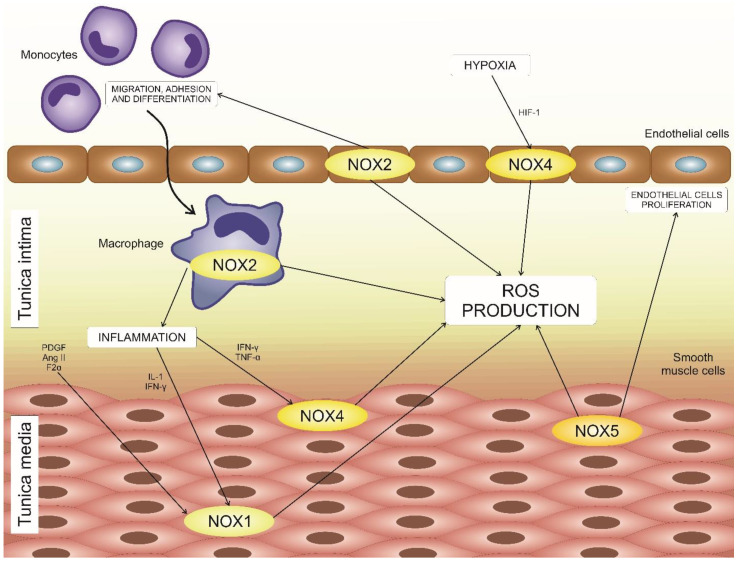
NOX isoforms expressed in vascular cells. NOX1, NOX2, NOX4, and NOX5 can be found in human vascular wall tissue. NOX1, NOX4, and NOX5 are expressed by vascular smooth muscular cells, while NOX2 and NOX4 are present in the endothelium. Macrophages recruited to the subendothelial cells also express NOX2. Together, these enzymes contribute to reactive oxygen species (ROS) formation in the vascular wall and play an important role in the development of local inflammatory processes.

Out of the six identified mammalian NOX family members, four are expressed in the vascular system cells (Figure 1). It was demonstrated that NOX1, NOX2, NOX4, and NOX5 are expressed in vascular smooth muscle cells (VSMC), endothelium, fibroblasts, and perivascular adipocytes. The remaining isoforms have not been detected or were found to be expressed at very low levels and their significance could not be estimated [5].

## 2. NOX Family Members of the Vascular System

### 2.1. NOX1

In humans, NOX1 is expressed in a wide variety of cells and tissues, including cells of the cardiovascular system, such as endothelial cells and vascular smooth muscular cells [6,7]. Moreover, NOX1 expression can be induced in some cell types by certain growth factors and signaling molecules. For instance, in VSMCs, NOX1 expression is triggered by platelet-derived growth factor (PDGF) and by the activity of angiotensin II and prostaglandin F2α [8,9]. Moreover, NOX1 is upregulated in the vascular wall under pathological conditions associated with inflammation, and proinflammatory molecules, such as interleukin-1 (IL-1) and interferon (IFN), mediate this process [10,11].

The implication of NOX1 in cardiovascular pathologies is relatively well documented. Studies in animal models have shown that vascular NOX1 expression appears to be important for hypertension, since overexpression of this enzyme was observed in different hypertension models (two-kidney two-clip renovascular hypertensive rats, transgenic hypertensive rats overexpressing the renin 2 gene, angiotensin II-infused mice, and others) [12,13].

The NOX1 promoter has various binding sites for transcription factors, such as members of the CREB/ATF family, NF-kB, AP-1, or Janus kinase/signal converters and transcription activators (JAK/STAT) [14]. In addition, due to the presence of elements rich in adenylate-uridylate (ARE), which are involved in degradation of mammalian mRNAs, post-transcriptional regulation of NOX1 mRNA via the 3’UTR is also possible.

### 2.2. NOX2

As mentioned above, NOX2 was historically the first NOX NADPH oxidase to be characterized. Oxidative bursts occurring in innate immune cells in response to pathogen invasion has been known for a long time. Initially, NOX2 was cloned and identified as phagocytic respiratory burst oxidase expressed by neutrophils and macrophages, which is critical for the initial nonspecific immune defense [1]. This enzyme, which later turned out to be the most widely expressed vascular NADPH oxidase isoform, was originally named gp91phox. Interestingly, the rapid increase of NOX2 activity needed for pathogen killing is facilitated by its transport from the intracellular compartments, where the inactive enzyme is stored, to the plasma membrane of activated cells. However, the functions of NOX2 are not limited to the host defense, and it can be activated in response to various stimuli in a wide range of cell types. The subcellular distribution of NOX2 is also cell-type-dependent.

In vascular ECs, NOX2 is involved in the regulation of endothelial functions, including the bioavailability of nitric oxide (NO), and modulation of the expression of adhesion molecules during inflammation and angiogenesis. Furthermore, NOX2 is also expressed in VSMCs, adventitious fibroblasts, and perivascular adipocytes [15].

It was found that NOX2 consists of the following subunits: gp91phox, p22phox, p47phox, p67phox, p40phox, and GTPase Rac1. The subunits gp91phox and p22phox are associated with the membrane and together form cytochrome b558, which is located in the cytoplasmic vesicles and the plasma membrane. Despite the fact that the structure of this NADPH oxidase is similar in different cell types, additional and regulatory subunits may be present in different cells under certain conditions [16]. For instance, the organizer protein NOX1 (NoxO1) and the activator protein NOX1 (NoxA1) were originally identified as regulators of NOX1 (instead of p47phox and p67phox, respectively). These subunits may also have moderate NOX2-activating properties. In the ECs, ROS generated by NOX2 are important for p38 MAP kinase-mediated proliferation and migration induced by vascular endothelial growth factor (VEGF) [17].

### 2.3. NOX4

Because of its structural features, NOX4 is capable of producing H_2_O_2_ directly [18]. Modulation of the activity of this enzyme is performed primarily at the level of its expression. For example, hypoxia induces the expression of NOX4 in SMCs of the pulmonary artery, while TGF-β induces NOX4 in cardiomyocytes and VSMCs, which was shown to promote vascular cells’ migration [19,20]. By contrast, PDGF, thrombin, and ligands activated by the peroxisome proliferator (PPAR) isoforms reduce the expression of NOX4 in VSMCs and ECs [21]. The promoter region of NOX4 gene contains many GC bases, which is characteristic for housekeeping genes, and indicates that NOX4 might itself be a housekeeping gene. The transcription factor E2F1 is implicated in the basal expression of NOX4 in rodent VSMCs. Basal expression of NOX4 was shown to be dependent on Sp3 and three GC-boxes, with the NOX4 gene containing putative Sp/Klf binding sites. In addition, in human ECs, basal transcription of NOX4 is regulated enzymatically by deacetylation of the transcription factor(s) and polymerase [22,23].

The JAK/STAT and NF-kB signaling pathways were shown to induce the expression of NOX4 in response to IFN-γ or TNF-α [24]. Moreover, hypoxia induces NOX4 through a mechanism that is dependent on factor-1 hypoxia (HIF-1). This factor contributes to maintaining the level of ROS in the SMCs of the pulmonary artery [25]. Unlike activation, the mechanisms of NOX4 inhibition have been insufficiently studied to date. A member of the AP-1 transcription factor family, JunD, which is known to prevent oxidative stress, was also shown to influence NOX4 expression. It was found that vascular expression of NOX4 was enhanced in JunD knockout mice [26]. More studies are needed, however, to map out the inhibitory pathways of NOX4 that may prove to be useful for treatment of conditions associated with oxidative stress.

### 2.4. NOX5

Four different NOX5 isoforms (α, β, γ, and δ) were found to be expressed in cultured human aortic SMCs, where they play regulatory functions. Overexpression of NOX5β or NOX5ε was shown to increase the ROS levels. However, the addition of calcium ionophores increased only ROS generated by NOX5β, but not NOX5ε [27]. NOX5-L and NOX5-S were shown to induce in vitro angiogenesis and proliferation in EC lines. Although NOX5ε was not found to be expressed in healthy coronary arteries, it was detected in coronary arteries of patients with coronary heart disease. It is possible that NOX5α and β are ROS-producing enzymes, and that NOX5ε may serve as a negative regulator of NOX5 [28]. The different splice variants of NOX5 have different location, activation, and modulation, which potentially makes NOX5 an important player of diverse signaling pathways that take place in the vasculature [29,30].

Together, the accumulated observations highlight the important role of NOX5 in the vasculature. NOX5-mediated ROS production is involved in signaling pathways that regulate the number of platelets, growth of SMCs, and capillary formation via the JAK/STAT signaling pathway. While depletion of NOX5 stimulates thrombin formation, its overexpression enhances endothelial proliferation and tubule formation [28].

## 3. Atherosclerosis: A Brief Overview of Pathogenesis

Atherosclerosis with associated cardiovascular and acute neurological conditions is the leading cause of morbidity and mortality worldwide. The hallmark of atherosclerosis is the formation of lipid-containing atherosclerotic plaques in the blood vessel wall that can obstruct the vessel lumen or undergo erosion or rupture, initiating further thrombotic events [31]. Atherosclerosis is known to be a complex pathology. Among the mechanisms involved in its initiation and progression are inflammatory processes, oxidative stress, and dysfunction of many cell types, enzymes, receptors, signaling molecules, and pathways [32]. Lipid accumulation in the arterial wall is mediated by low-density lipoprotein (LDL) internalization by the arterial wall cells, both resident and recruited from the circulation, such as monocytes/macrophages [33].

Inflammation plays a central role at all stages of atherosclerosis development. Activation of the arterial endothelium is considered as the earliest event in the atherosclerotic plaque formation. Such activation can occur in response to mechanical or biochemical stimuli, but the possible role of pathogens has also been discussed. Local activation of the ECs is accompanied by the expression of adhesion molecules and the recruitment of innate immunity cells to the future lesion site. Monocytes/macrophages enter the subendothelial space of the arterial wall intima, where they actively participate in intracellular lipid accumulation by engulfing LDL particles. Together with the resident intimal cells, macrophages accumulate lipoproteins in the cytoplasm, being unable to digest them in a normal way, which requires controlled, receptor-mediated endocytosis rather than uncontrolled phagocytosis. Cells with cytoplasm filled with lipid droplets are called foam cells and represent an abundant cellular component of growing atherosclerotic plaques. The appearance and growth of atherosclerotic plaques is therefore caused by the immunological interaction between subsets of innate immunity cells, ECs, and resident VSMCs of the arterial wall [32,34]. Moreover, extracellular matrix remodeling is also involved into the development of atherosclerotic plaques. Thus, impaired recovery and thinning of the fibrous cap and erosion of plaque can be caused by changes in collagen metabolism. Degradation of extracellular matrix components, for example, by matrix metalloproteinases (MMPs) was associated with plaque rupture and superimposed thrombosis [35].

The link between cholesterol accumulation in the arterial wall and atherosclerosis development is currently well established. Cholesterol is carried by LDL, and high levels of LDL cholesterol in the blood are closely associated with coronary artery disease. In blood plasma, numerous modifications of LDL particles take place, giving rise to more atherogenic LDL forms. LDL particles can undergo changes of size and density, acquire negative electric charge, and undergo desialylation, oxidation, and other chemical modifications. These atherogenic modifications of LDL are important for the pathogenesis of the disease [36]. Moreover, such modified LDL can be an important stimulator of the immune response, contributing to the inflammatory pathways of atherosclerosis development [37]. Despite the fact that lipids and the immune system are considered to be the main determinants of atherosclerosis, many pathways that facilitate cross-talk between lipids and immune cells have not yet been identified. The modulation of the immune system response under conditions of chronic hyperlipidemia appears to be an important early disease pathway.

Over the past decade, clinical atherosclerosis treatment has been mostly focused on lowering the lipid level. Despite the fact that our understanding of cholesterol metabolism and the development of lipid-lowering drugs, in particular 3-hydroxy-3-methylglutaryl-CoA reductase inhibitors (statins), helped reducing the incidence of cardiovascular diseases, the problem cannot be considered as resolved. Future efforts are likely to focus on the inflammatory component of the disease pathogenesis. The development and testing of new anti-inflammatory approaches is a key component in the development of novel antiatherosclerosis therapies, as well as for the treatment of cardiovascular disease, in addition to optimal lipid-modulating therapy. It is assumed that specific immunomodulatory therapy may not only restore the impaired immune response, but also correct dyslipidemia [31,38].

Statins are currently widely used to lower plasma LDL cholesterol. This kind of therapy is effective, as it has shown the ability of inhibiting inflammatory processes and reducing the content of macrophages in the lesion [39]. Besides, statins have also been shown to possess pleiotropic activity and to have anti-inflammatory effects, especially relevant for cardiovascular disease [40]. Currently, one of the key unanswered questions is whether the long-term anti-inflammatory effect of statins is a consequence of their hypolipidemic effect or a consequence of inhibition of the mevalonate pathway. In this regard, it would be interesting to determine the effect of strong lipid-lowering treatment of anti-PCSK9 on the inflammatory status of patients. Various clinical trials are currently underway to detect mild inflammation in patients with cardiovascular disease, as well as with elevated levels of IL-1, IL-6, and highly sensitive reactive protein C (hsCRP) [32,41].

## 4. NADPH Oxidases in Atherosclerosis Development

The implication of upregulation of NADPH oxidases was shown to be involved in cardiovascular disease development in numerous studies. With regard to atherosclerosis, the most important disease processes may be referred, at least in part, to NADPH oxidases functioning, including oxidative stress, vascular inflammation, endothelial dysfunction, and vascular remodeling [42].

Oxidative stress is currently regarded as one of the important components of atherosclerosis pathogenesis. One of its consequences is oxidative modification of LDL [43]. Moreover, it was shown that ROS generated by NADPH oxidase activity are early inducers of autophagy, which serves as a potential link to NADPH oxidases as the main ROS source [44]. Different NOX isoforms are detected in various cell types implicated in atherogenesis. Thus, ROS-producing NOX2 was found in the endothelium and the adventitia, while NOX1 and NOX4 were shown to be important for the functioning of VSMCs [45].

The regulatory activity of NADPH oxidases can be relevant at early stages of atherosclerosis development. For instance, Rac, RhoA, paxillin, cofilin, and other proteins participating in cell adhesion were demonstrated to be activated by NOX1. Its levels in atherosclerotic lesions of both humans and rabbits were shown to be extremely low, while overexpression of NOX1 was observed in patients with cardiovascular disease or diabetes mellitus [46,47]. At the same time, overexpression of NOX1 contributed to enhanced neointima formation upon vascular injury, indicating that this enzyme may be involved in regulating cell proliferation in the arterial wall [48]. However, the data available so far are controversial and do not allow making any strong suggestions about the role of NOX1 in atherosclerosis development.

NADPH-oxidases-dependent ROS formation in the ECs and VSMCs can serve as a potent activator of the expression of adhesion molecules and the subsequent monocyte/macrophage infiltration into the arterial wall. Moreover, they contribute to endothelium activation and stimulate VSMCs proliferation. The impact of different NOX isoforms has been studied in knockout animal models of atherosclerosis. It was demonstrated that NOX2 was not an atherosclerosis inducer in apolipoprotein E knockout (*apoE*-KO) mice, which are prone to atherosclerosis development. However, endothelial-specific overexpression of NOX2 was capable of enhancing superoxide generation, endothelial vascular cell adhesion molecule-1 expression, and recruitment of macrophages [49,50]. Despite the lack of information, some promising approaches to use NOX2 as a therapeutic target have already been tested, such as the recently demonstrated atheroprotective effect on NOX2 of salvianic acid A [51].

Among the NADPH oxidases, NOX4 is currently the most studied in relation to atherosclerosis, as it is believed to have atheroprotective properties. In place of superoxide, this enzyme releases another ROS precursor, hydrogen peroxide, due to the spontaneous superoxide dismutation. Hydrogen peroxide does not interact with NO, so NOX4 does not cause peroxynitrite generation [18,52]. Moreover, hydrogen peroxide released by NOX4 is also capable of lowering the VSMC proliferation rate, preventing vascular remodeling and inflammation and maintaining eNOS and heme oxygenase-1 expression in the setting of vascular stress [53,54]. In contrast to these benefits, detrimental effects of NOX4 were demonstrated in several rodent models, among which were animals with cardiac hypertrophy and ischemic stroke [55]. Hydrogen peroxide, as well as other ROS, can act both as a protective and damaging agent, which depends on the cell type expressing the NOX enzyme, the amounts of released compound, and its subcellular location.

NOX5 expression was detected during the early stages of the endothelial lesion development and in VSMCs of the intima of advanced coronary lesions. Calcium-dependent NOX5 is an important source of ROS in atherosclerosis, which makes it also important for oxidative damage. In the coronary arteries from patients with coronary heart disease, the levels of NOX5 RNA and protein were demonstrated to be significantly higher as compared to healthy arteries, which was also consistent with the Ca^2+^-dependent activity of NADPH oxidase in arteries [56].

## 5. The Effects of NADPH Oxidases on Vascular Remodeling

**Figure 2:**
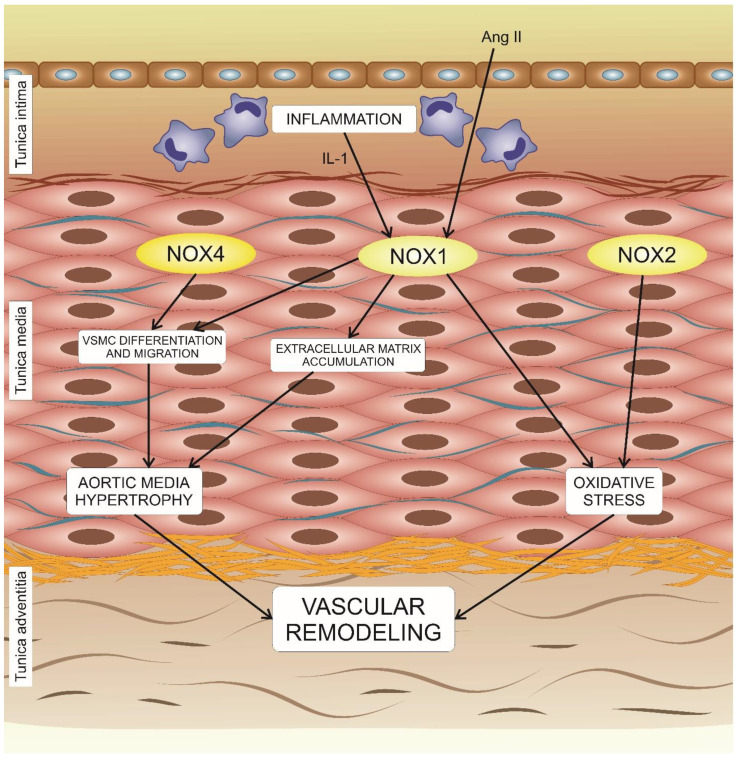
Role of NOX isoforms in vascular remodeling. Oxidative stress and hypertrophy of aortic media in response to angiotensin II (Ang II) signaling and inflammation are mediated by several isoforms of NOX and together lead to vascular remodeling processes.

Based on the available data from different studies, it was postulated that NOX enzymes are required for vascular remodeling in different cardiovascular disorders, including atherosclerosis. However, the source(s) and regulation of NOX-dependent ROS in vascular remodeling currently remain to be studied in detail. The significance of NOX1 for vascular remodeling was reported by several studies. In response to angiotensin II (AngII), mice with knockdown of *nox1* developed a significant decrease in aortic medium hypertrophy. However, the reason was not the decreased vascular smooth muscular cell count, since AngII-induced proliferation of cells was preserved, but the extracellular matrix accumulation. It was also demonstrated that AngII together with IL-1 stimulate NOX1-dependent migration of VSMCs (Figure 2) [11,57]. Numerous studies aimed to characterize the relationship of AngII and NOX1 with the cardiovascular risk, but the role of NOX1 in small artery remodeling was not the focus. These studies, however, established that the cell-specific location of NOX1 might be the key to modulating hypertrophic vascular remodeling. In VSMCs, NOX1 appeared to be of fundamental importance [13,58]. In 2018, Vendrov and coauthors reported the results of the study that involved both genetic mouse models and cell culture study, according to which NOX1 coactivator protein, NoxA1, critically regulates smooth muscle growth, migration, and phenotypic modulation in stenotic and atherosclerotic vascular remodeling [59].

Another NADPH oxidase that appears to be important for vascular remodeling is NOX4. It was shown that NOX4 affects a wide range of cellular processes associated with remodeling, among which are cell proliferation, apoptosis, senescence, cell differentiation, cell migration, and cell cycle regulation. It is illustrated, for example, by reversing of 7-ketocholesterol-induced apoptotic events by silencing NOX4 expression [60].

The role of NOX2 in adventitial vascular remodeling was evaluated in several studies [61]. A recent study reported that the AIp1 protein, the key negative regulator of NOX2, could provide a new mechanism for NOX2 regulation by suppressing NOX2-dependent oxidative stress in the vasculature to modulate vascular remodeling [62]. More studies are needed, however, to outline the NOX-mediated pathways in vascular remodeling and to identify relevant therapeutic targets.

## 6. Available Therapies and Future Perspectives

Despite the wide recognition of oxidative stress as one of the key pathogenesis components and potential points of therapeutic intervention, development of specific antioxidant therapies that would be effective for treatment of atherosclerosis remains the subject of future research. The use of nonspecific antioxidant therapies could not be proven effective in clinical studies conducted. More specific agents that can target oxidative stress at the local level of the atherosclerotic lesion appear to be more promising. Among them, NOX inhibitors can be listed as attractive potential antiatherosclerotic drugs. Several molecules that affect NOX in an unspecific manner are known, including some drugs that are commonly used for treatment human disorders, such as angiotensin receptor inhibitors and statins [63,64]. However, these agents can inhibit both pathological and physiological activity of NOX.

The development of more selective, isoform-specific NOX inhibitors is currently underway. Two small molecules, GKT136901 and GKT137831, are selective for NOX1 and NOX4 isoforms. The agents have been tested in a range of animal disease models, including *apoE^−/−^* mice [65] and a murine hypoxia model [66]. These inhibitors were able to selectively reduce ROS formation in the aorta and alleviate inflammation and macrophage recruitment in the arterial wall. Subsequently, GKT137831 has been tested in clinical trials and was shown to have good tolerability combined with anti-inflammatory effect, which was promising for the development of future antiatherosclerosis therapies [67]. Agents selectively targeting NOX2 have also been tested in animal models. Tempol, a superoxide scavenger, has been shown to attenuate oxidative stress and atherosclerosis in an animal model of metabolic syndrome by interfering with NOX2 complex assembly and activity [68]. A recent study demonstrated that selective pharmacological inhibition of NOX2 with a selective inhibitor, gp91dstat (as opposed to genetic deletion), protected *apoE^−/−^* mice with EC-specific insulin resistance against oxidative stress and vascular damage, and alleviated superoxide production in human saphenous vein endothelial cells [68]. More studies are required, however, to translate these interesting findings to clinical practice.

The results available so far allow outlining several potential avenues for future research on NADPH-oxidase-targeting therapies. First, more studies are needed to better characterize the role of each isoform in the pathology development to identify the points of therapeutic intervention with better precision. It is clear that complete and permanent inhibition of even one of the NOX isoforms may not only be ineffective, but aggravate atherosclerosis development [69]. It is likely that different NOX isoforms become activated in certain tissues and at certain timepoints of pathology development, otherwise playing an important physiological role that should be preserved by therapies. Second, the search for isoform-specific NOX inhibitors should be continued. So far, only a few small molecules have been characterized, while the majority of known NOX inhibitors are peptides. That requires development of innovative delivery methods in order to use these agents for drug development. Finally, the identified selective NOX inhibitors that have shown promising results in preclinical studies should be tested clinically in relevant subpopulations of patients presenting with cardiovascular disease risk factors, such as chronic inflammation, metabolic syndrome, hyperlipidemia, or diabetes.

## 7. Conclusions

Different aspects of NADPH oxidases’ impact on atherosclerosis have been evaluated and described. NOX1, NOX2, NOX4, and NOX5 appear to be of particular interest for atherosclerosis, but, unfortunately, data which demonstrate the particular functions and their mechanisms are disparate. Thus, the NOX1 level in atherosclerotic lesions in both humans and rabbits was shown to be extremely low, while its overexpression was observed in patients with cardiovascular disease or the development of diabetes mellitus. NOX2 was shown to be a crucial source of ROS involved in vascular remodeling, while NOX5 was also considered to be a major ROS source due to the fact that its expression was detected in the early stages of the endothelial lesion and in smooth muscular cells of the intima in advanced coronary lesions. NOX4 was shown even to have an atheroprotective effect because this enzyme releases not superoxide, but another ROS precursor, hydrogen peroxide, due to the spontaneous superoxide dismutation within the NOX4 enzyme. However, further studies discovered that NOX4 also has a detrimental effect in several animal models of cardiovascular disorders. There is currently little doubt that NADPH oxidases are implicated in a variety of processes relevant to atherogenesis, such as oxidative stress, vascular inflammation, endothelial dysfunction, and vascular remodeling. Several pharmacological agents selectively inhibiting NOX isoforms are being tested preclinically and clinically, and they have already delivered some promising results for treatment of atherosclerosis. However, molecular mechanisms through which NADPH oxidases participate in atherosclerosis are still insufficiently understood and need to be investigated further. The search for novel NADPH oxidase inhibitors suitable for drug development should also continue.
